# Leflunomide as a Corticosteroid-Sparing Agent in Giant Cell Arteritis and Polymyalgia Rheumatica: A Case Series

**DOI:** 10.1155/2013/120638

**Published:** 2013-09-11

**Authors:** Andreas P. Diamantopoulos, Helene Hetland, Geirmund Myklebust

**Affiliations:** ^1^Department of Rheumatology, Hospital of Southern Norway Trust Kristiansand, Service Box 416, 4604 Kristiansand, Norway; ^2^Faculty of Medicine, Norwegian University of Science and Technology, Service Box 8905, 7491 Trondheim, Norway

## Abstract

*Objectives*. Giant cell arteritis (GCA) and polymyalgia rheumatica (PMR) affect individuals older than 50 years of age and corticosteroids are the mainstay of treatment. The aim of our study was to explore the role of leflunomide as a corticosteroid-sparing agent in GCA and PMR patients. *Methods*. Patients with difficult-to-treat GCA and PMR were retrospectively identified in the period from 2010 to 2013. The doses of corticosteroids and CRP values were noted before, after three months, and at the end of the treatment with leflunomide (for patients continuing treatment, censoring date was January 1, 2013). *Results*. Twenty-three patients were identified (12 with PMR and 11 with GCA). A reduction of 6 mg/dL (CI 95% –10.9–34.2, *P* = 0.05) in CRP and 3.7 mg (CI 95% 0.5–7.0, *P* = 0.03) in prednisolone dose was observed in the PMR group. In GCA patients, the reduction was 12.4 mg/dL (CI 95% 0.7–25.5, *P* = 0.06) in CRP and 6.6 mg (CI 95% 2.8–10.3, *P* < 0.01) in prednisolone dose. *Conclusion*. Leflunomide seems to be effective as a corticosteroid-sparing agent in patients with difficult-to-treat GCA and PMR. Randomized controlled trials are warranted in order to confirm the usefulness of leflunomide in the therapy of GCA/PMR.

## 1. Introduction

GCA and PMR affect individuals older than 50 years of age and corticosteroids are the mainstay of treatment [1, 2]. However, there is an unmet medical need of alternatives in the treatment of the GCA and PMR as 30% of GCA and 50% of PMR patients will relapse [3] or have difficulties to reduce the corticosteroid doses. In addition, long-term toxicity is a well-known side effect of corticosteroids [3]. Azathioprine in GCA [4] and methotrexate in GCA and PMR [3, 5] have been tested in randomized control trials and have shown low to moderate efficacy as corticosteroid-sparing agents [6]. 

Leflunomide, a pyrimidine synthesis inhibitor, is approved as a disease-modifying antirheumatic drug in the treatment of rheumatoid arthritis (RA) [7] and psoriatic arthritis (PsA) [8]. In vasculitides, leflunomide has shown efficacy in the treatment of Takayasu's arteritis [9], granulomatosis with polyangiitis (GPA) [10], and a case series of GCA and PMR patients [11]. It seems that leflunomide inhibits the dendritic cell maturation [12] and the production of IL-17 [13] which are both involved in pathogenesis of GCA and PMR [14].

The aim of this retrospective case series was to study the role of leflunomide as a corticosteroid-sparing agent in difficult-to-treat GCA and PMR patients.

## 2. Methods

Patients with difficult-to-treat GCA and PMR were retrospectively identified in case records of the Vasculitis clinic at the Hospital of Southern Norway for the period from 2010 to 2013. To enter the study, the patients had to have difficult-to-treat disease or suffer a flare either when reducing corticosteroids or on treatment with methotrexate. Prednisolone dose had to be >5 mg daily. The flare and the difficult-to-treat disease were defined as follows:

1. for PMR persistent or relapsed pain in the shoulder or hip girdle and/or elevated CRP,
2. for GCA, persistent or relapsed cranial symptoms (headache, jaw claudication), vision disturbances (amaurosis fugax, diplopia), pain in the shoulder or hip girdle, and/or increased CRP.

All the patients fulfilled either the ACR classification criteria for GCA [15] or the EULAR/ACR classification criteria for PMR [16]. The patients started up with 10 mg leflunomide and the dose was escalated up to 20 mg if the clinical response was insufficient or according to the judgment of the treating physician. The doses of corticosteroids and CRP values were noted before, after 3 months, and at the end of the treatment (for patients continuing treatment, censoring date was January 1, 2013). Side effects were also registered. The criteria for remission were a symptom-free patient with prednisolone dose lower than 2.5 mg.

The patients who discontinued treatment due to side effects or remission were included in the data analysis.

### 2.1. Statistical Analysis

Student's *t*-test was used to compare the means and Wilcoxon Signed-Rank test was used for the nonparametric numerical variables. The Chi-square test was used to compare categorical variables. All the statistical analyses were performed by using the SPSS statistical package version 17 (SPSS Inc. Chicago IL, USA). Statistical significance was defined as *P* < 0.05. 

### 2.2. Ethics Statement

The study was approved by the Institutional Review Board, Hospital of Southern Norway Trust. Due to the retrospective design of the study, informed consent has not been obtained. The data were analyzed anonymously.

## 3. Results

Twenty-three patients were identified (3 males, 20 females, mean age 69 years), 12 with PMR and 11 with GCA during the observation period. The baseline characteristics of the patients are presented in Table 1.

Six patients (26%) (3 PMR and 3 GCA) discontinued treatment due to side effects and 5 patients (21%) (2 PMR and 3 GCA) due to remission. The remission was achieved in a mean period of 10.2 months (CI 95% 3.7–16.7). The most common adverse event was diarrhea (3 patients) followed by rash (2 patients). One patient discontinued treatment due to general malaise. No serious adverse events requiring hospitalization were recorded. 

### 3.1. PMR Patients

The mean duration of treatment was 10.5 months (CI 95% 4.7–16.3) for the PMR patients. A reduction of 6 mg/dL in the CRP values (CI 95% 10.9–34.2, *P* = 0.05) and 3.7 mg (CI 95% 0.5–7.0, *P* = 0.03) in the prednisolone dose was observed in the PMR group. The reduction of prednisolone dose was 34.2% for the PMR patients. At three-month treatment with leflunomide, no statistical significant difference was seen in prednisolone dose or CRP values. 

### 3.2. GCA Patients

The mean duration of treatment was 10.9 months (CI 95% 3.7–18.3) for the GCA patients. The reduction in CRP values was 12.4 mg/dL (CI 95% 0.7–25.5, *P* = 0.06) and prednisolone dose 6.6 mg (CI 95% 2.8–10.3, *P* < 0.01). The reduction of prednisolone dose was 63.4% for the GCA patients. A statistically significant reduction (14.5 mg/L) in CRP (CI 95% 2.1–26.8, *P* = 0.02) and 3.8 mg in prednisolone dose was observed already after three months of treatment (CI 95% 0.6–6.9, *P* = 0.02).

## 4. Discussion

The main finding of our study is that leflunomide seems to be effective as a corticosteroid-sparing agent in patients with difficult-to-treat GCA and PMR. To our knowledge, this is the first study showing a significant corticosteroid-sparing effect of leflunomide in GCA and PMR. Furthermore, it appears that patients suffering from GCA respond better to treatment with leflunomide by achieving a greater reduction in prednisolone dose (63.4% versus 34.2%) than PMR patients. In addition, in GCA patients the sparing effect seems to be present after a relatively shorter time of leflunomide treatment than in PMR. 

In our study, we observed a reduction in the inflammation markers (CRP) in both PMR and GCA. However, this reduction reached a statistically significant level only in the PMR group of patients. 

In our cohort, a significant part of patients (23%) went into remission after treatment with leflunomide in a mean of 10.2 months. The corticosteroid-sparing effect of leflunomide was achieved with a low dose of 12.7 mg in GCA and 14 mg in PMR. The recommended loading dose of 100 mg in treatment of arthritides was not used due to the fact that such a high dose is often associated with side effects. In other autoimmune diseases, doses of 20 mg (RA, PsA) [5, 6] and 30 mg (GPA) [8] were used in order to induce remission.

A high rate of withdrawals was observed. One in 4 patients discontinued leflunomide treatment due to side effects. A high rate of dropouts has been reported in leflunomide treatment in other diseases [7, 15]. However, in the present study no serious side effects requiring hospitalization were observed and all patients recovered after the withdrawal of leflunomide. Interestingly, no patients reported frequent infections during leflunomide treatment.

Our study has some limitations. In a retrospective collection of data, some information about the side effects and the dosage of prednisolone or the recorded CRP values could be missing. However, no such data was lacking in our cohort. The small number of patients, the short time of followup, and the absence of control group are also weaknesses of our study. 

In conclusion, leflunomide seems to be effective as a corticosteroid-sparing agent in difficult-to-treat GCA and PMR patients. Randomized controlled trials are warranted to confirm the usefulness of leflunomide in the treatment of GCA/PMR.

## Figures and Tables

**Table 1:** Baseline characteristics of the enrolled patients.

	PMR (#12)	GCA (#11)	*P* values
Mean age in years (SD)	67.4 (7.5)	71.1 (6.0)	0.21
Median disease duration in months (range)	14 (4–78)	20 (4–120)	0.50
Number of patients used methotrexate before (%)	3 (25.0)	7 (63.6)	0.06
Median prednisolone dose in mg (range)	10.5 (5–17)	10.4 (7–20)	0.95
Median CRP in mg/L (interquartile range)	9.5 (26.2)	12.0 (24)	0.70
Mean leflunomide dose in mg (CI 95%)	14 (10.8–17.4)	12.7 (9.5–15.8)	0.49

PMR: polymyalgia rheumatica, GCA: giant cell arteritis, SD: standard deviation, CRP: C-reactive protein, CI: confidence interval, #: number of patients enrolled.
